# A novel smartphone app for blood pressure measurement: a proof-of-concept study against an arterial catheter

**DOI:** 10.1007/s10877-022-00886-2

**Published:** 2022-06-21

**Authors:** G. Hofmann, M. Proença, J. Degott, G. Bonnier, A. Lemkaddem, M. Lemay, R. Schorer, U. Christen, J.-F. Knebel, P. Schoettker

**Affiliations:** 1 Department of Anesthesiology, Lausanne University Hospital and University of Lausanne, Lausanne, Switzerland; 2 CSEM, Centre Suisse d’Électronique et de Microtechnique, Neuchâtel, Switzerland; 3 Department of Anesthesiology, Geneva University Hospital and University of Geneva, Geneva, Switzerland; 4 Biospectal SA, 1003 Lausanne, Switzerland

**Keywords:** Arterial hypertension, Mobile phone, Mobile health, Trending ability, Smartphone, Blood Pressure

## Abstract

Smartphones may provide a highly available access to simplified hypertension screening in environments with limited health care resources. Most studies involving smartphone blood pressure (BP) apps have focused on validation in static conditions without taking into account intraindividual BP variations. We report here the first experimental evidence of smartphone-derived BP estimation compared to an arterial catheter in a highly dynamic context such as induction of general anesthesia. We tested a smartphone app (OptiBP) on 121 patients requiring general anesthesia and invasive BP monitoring. For each patient, ten 1-min segments aligned in time with ten smartphone recordings were extracted from the continuous invasive BP. A total of 1152 recordings from 119 patients were analyzed. After exclusion of 2 subjects and rejection of 565 recordings due to BP estimation not generated by the app, we retained 565 recordings from 109 patients (acceptance rate 51.1%). Concordance rate (CR) and angular CR demonstrated values of more than 90% for systolic (SBP), diastolic (DBP) and mean (MBP) BP. Error grid analysis showed that 98% of measurement pairs were in no- or low-risk zones for SBP and MBP, of which more than 89% in the no-risk zone. Evaluation of accuracy and precision [bias ± standard deviation (95% limits of agreement)] between the app and the invasive BP was 0.0 ± 7.5 mmHg [− 14.9, 14.8], 0.1 ± 2.9 mmHg [− 5.5, 5.7], and 0.1 ± 4.2 mmHg [− 8.3, 8.4] for SBP, DBP and MBP respectively. To the best of our knowledge, this is the first time a smartphone app was compared to an invasive BP reference. Its trending ability was investigated in highly dynamic conditions, demonstrating high concordance and accuracy. Our study could lead the way for mobile devices to leverage the measurement of BP and management of hypertension.

## Introduction

Worldwide, hypertension is one of the largest contributor to heart disease and stroke and the leading risk factor responsible for ten million premature deaths [1, 2]. With a current trend showing an increase of > 500 million people since the 1970s, prevalence is expected to increase to 1.56 billion worldwide by 2025 [3], driven largely in low and middle-income countries (LMIC) [4]. Accurate, reliable and repeated blood pressure measurements are essential for the diagnosis of hypertension. In this context, out-of-office blood pressure has emerged as an effective, affordable and convenient means of screening [5] with better prognostic ability than office blood pressure monitoring [6, 7].

Mobile revolution applied to healthcare is a promising candidate to leverage out-of-office blood pressure monitoring as numerous systematic reviews and meta-analyses have enforced the effectiveness of mobile health to improved medical adherence by lowering both mortality and morbidity [8]. Hence, bringing to the populations a mobile application allowing them to measure accurately their blood pressure at any time would be an important step in the global health prevention strategy.

**Fig. 1:**
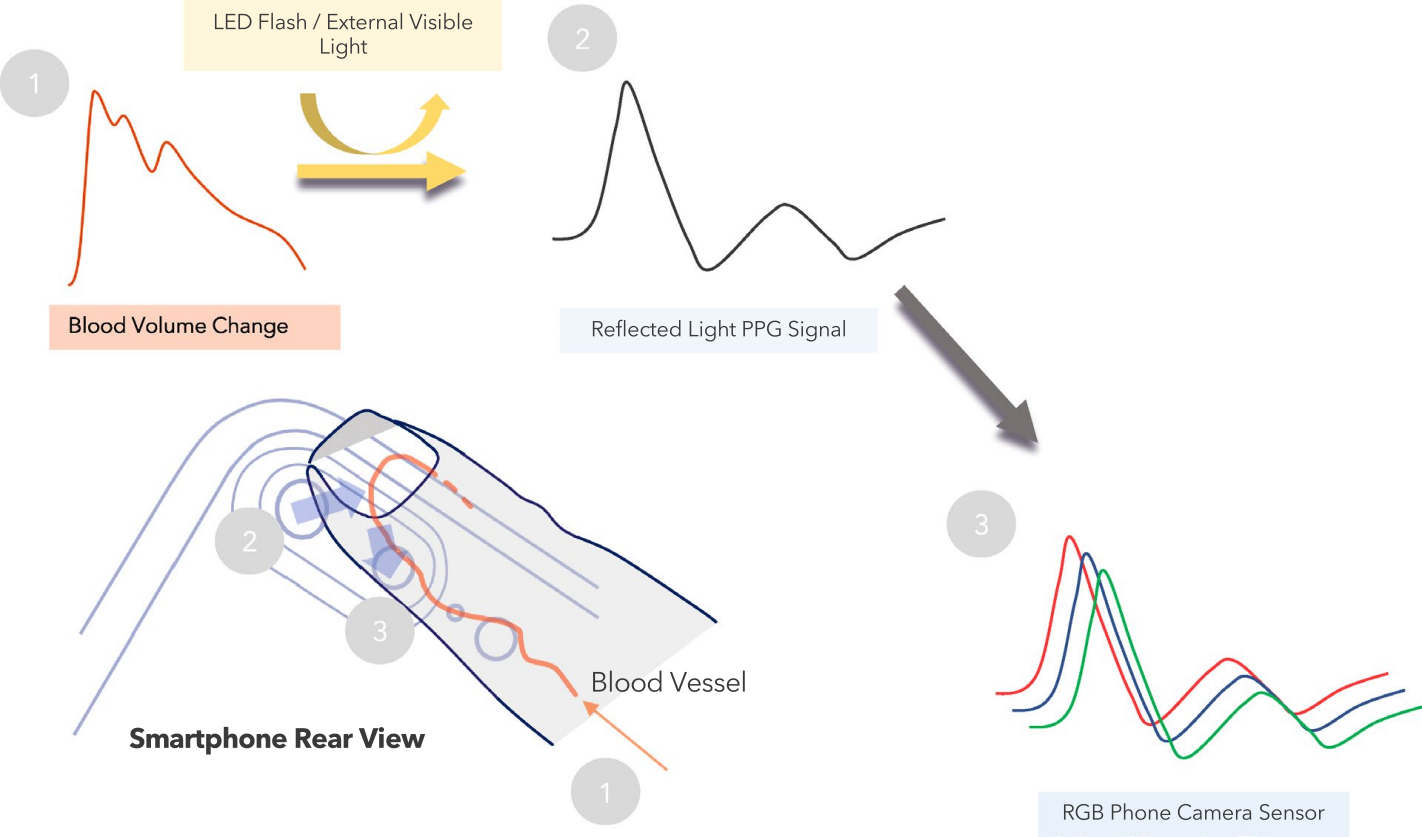
The app utilizes image data generated from volumetric blood flow changes (1) via light passing through the fingertip, reflecting off the tissue (2), and then passing to the phone camera’s image sensor (3)

In the last decade, the development of techniques based on the indirect measurement of pulse wave velocity using non-invasive techniques such as tonometry, photoplethysmography (PPG), and electrocardiography have opened new horizons for the cuff-less monitoring of blood pressure [9–11]. These solutions are typically based on the measurement of the pulse propagation time between two arterial sites [12] but technical constraints have prevented these models to become reliable clinical tools for measuring BP [9, 13]. Pulse wave analysis (PWA) relies on the biomechanical and physiological correlation between BP and the wave morphology along the arterial tree [14, 15] (Fig. 1). The resulting arterial pulses are the superposition of forward and backward waves interfering at various locations of the arterial tree. The clinical benefits of using PWA to determine BP compared to a cuff sphygmomanometer was demonstrated by The Conduit Artery Function Evaluation (CAFE) study [16] and the Strong Heart Study [17, 18]. In these two studies, central systolic and pulse pressures were more sensitive than peripheral brachial blood pressure to assess cardiovascular risk and pharmacological interventions. Recently, IPAAR Trial (iPhone App Compared With Standard RR Measurement) failed to validate a BP algorithm to estimate SBP recorded with a smartphone camera but emphasized and confirm the potential of a smart device to screen for hypertension in the general population in the future [19, 20].

In this context, a smartphone application based on PWA (OptiBP™, Biospectal, Switzerland) has been validated on a general population compared to a dual-head stethoscope and sphygmomanometer method as described in ISO 81060-2 standards, and in various other settings [21–24]. Although the ISO universal standard may validate the accuracy and precision of the above solution, its ability to track intra-patient BP variations remains to be validated [25].

The aim of our current study was to evaluate the blood pressure trending ability of the OptiBP app using a Pulse Wave Analysis compared to invasively acquired blood pressure measurements in a context of high BP variability such as induction of general anesthesia. The second and third part of our analysis aimed at assessing clinical concordance using error grid analysis as well as accuracy and precision based on the ISO 81060-2 standards for non-invasive sphygmomanometers.

## Methods

### Study design and population

For this prospective multicentric study, we obtained informed consent from 121 patients aged 18 years or more and scheduled for an elective surgery requiring general anesthesia and invasive BP monitoring at CHUV (University Hospital of Lausanne, Switzerland) and HUG (University Hospital of Geneva, Switzerland) between April 2019 and November 2019. Exclusion criteria were patient refusal, age below eighteen, inability to give informed consent, ASA risk > 3, dysrhythmia (bigeminy, trigeminy, isolated VPB, atrial fibrillation), contraindication to the placement of an arterial catheter (Raynaud’s disease, Burger arthritis, negative Allen test, major hyperlipidemia), or known contact dermatitis to nickel/chromium [26].

Patients were prepared for anaesthesia according to the existing safety and standard procedures of the Department of Anesthesiology of CHUV Lausanne and HUG Geneva, tailored individually to the patient, depending on his concomitant disease, treatments, and procedures. A dedicated catheter (BD Arterial Cannula 20G/1.1 mm × 45 mm, Becton Dickinson Infusion Therapy Syst. Inc., UT, USA) was inserted predominantly into the right or left radial artery under local anesthesia, allowing beat-to-beat continuous blood pressure monitoring. Patients were monitored with Philips® IntelliVue MP50 monitor conjointly with M3001A module for IAP measurements (Philips, Amsterdam, the Netherlands). General anesthesia was induced with an infusion of propofol (2–3 mg/kg) while intravenous analgesia was provided by boluses of fentanyl (1–2 μg/kg) or continuous remifentanil infusion (0.1–0.5 μg/kg/min) depending on the surgical intervention. Rocuronium (0.6 mg/kg) was administered before intubation. The management of the anesthesia was left at the discretion of the anesthesiologist in charge and maintenance provided with propofol (6–12 mg/kg/h). Boluses of ephedrine (5–10 μg) or phenylephrine (50–100 μg) depending on the clinical context were used in case of necessity for hemodynamic support.

The study was approved by the local ethics committee (CER-VD no 2018-01656) and registered under number NCT03875248 (Arm 1) at www.clinicaltrials.gov. The clinical investigation was conducted in compliance with the European Directive 93/42/EEC on medical devices [27] with the Swiss Ordinance on clinical trials of therapeutic products [28] and with international standards ISO 14155:2011 [29]. In the absence of standards applicable to our cuffless approach, we evaluated the performance of the app using the standards of the ISO 81060-2:2018 norm [30].

### Data processing and analysis

#### Invasive data processing

The continuous invasive BP was recorded at induction of general anesthesia for 20 min.

**Fig. 2:**
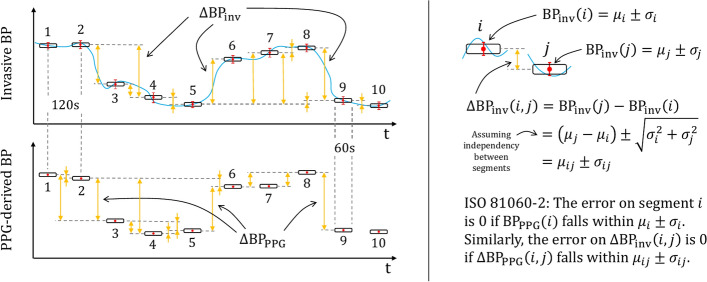
Identification of invasive BP changes (∆BP_inv_) and comparison with their corresponding PPG-derived BP changes (∆BP_PPG_). All possible pairs of BP changes between the ten recordings of each patient were considered; only a few of them are illustrated in the figure as orange arrows for readability reasons. *BP* blood pressure; *∆BP* BP change; *PPG* photoplethysmography; *BP*_*inv*_ invasive BP; *BP*_*PPG*_ PPG-derived BP

All data were recorded with the ixTrend express software version 2.1.0 (ixellence GmbH, Wildau, Germany) installed on a laptop computer connected to the monitor and analyzed and post-processed offline using MATLAB version R2020b (The MathWorks, Inc., Natick, USA). For each patient, ten 1-min segments aligned in time with ten smartphone recordings were extracted from the continuous invasive BP (BP_inv_) recording, as illustrated in Fig. 2. For each 1-min segment of invasive BP data, the average value and the standard deviation (SD) of SBP_inv_ (systolic), DBP_inv_ (diastolic) and MBP_inv_ (mean) were computed.

#### Smartphone data processing

We used a Samsung Galaxy S7 (Samsung GEC, 26, Sangil-ro 6-gil, Gagdong-gu, Seoul, Korea). Similarly, to the invasive BP data, each 1-min smartphone video recording was post-processed and analyzed in MATLAB to obtain a PPG-derived SBP_PPG_ (systolic), DBP_PPG_ (diastolic) and MBP_PPG_ (mean) value per recording. To that end, for each 1-min sequence of images acquired with the smartphone, the pixels from the green channel of the central region of each image in the video sequence were averaged to obtain a PPG signal. From each 1-min PPG signal, a BP estimate was obtained in a fully automated manner via our PWA algorithm [31] which maps morphological features of PPG waveforms into BP estimates via a non-linear model. In addition to providing BP estimates, the algorithm automatically rejects unreliable BP estimates obtained from PPG signals it considers of insufficient quality. The algorithm was previously described in detail and validated in pulse oximeter PPG signals during general anesthesia [32].

### Algorithm parameters training

The parameters $\widehat{\theta }$ of the non-linear model applied to the set of morphological features extracted by the algorithm were trained using data acquired by a previously published study [32], minimizing in the least-square sense the error between BP_inv_ changes (∆BP_inv_) and BP_PPG_ changes (∆BP_PPG_). To that end, significant changes in BP in the invasive reference data were selected and compared to their corresponding PPG-derived BP changes. By concatenating all selected ∆BP_inv_ changes and their corresponding ∆BP_PPG_ values for all 40 patients in vectors ${\varvec{U}}$ and ${\varvec{V}}$ respectively, the parameters $\widehat{\theta }$ of the model were optimized in the least-square sense, i.e., by solving $\widehat{\theta }={\underset{\theta }{\mathrm{argmin}} \Vert {\varvec{U}}-{\varvec{V}}\Vert }^{2}$. The thus trained model was then applied, with no further adaptation, to the smartphone-derived PPG data in the present study.

### Statistical analysis

The main part of our study focused on assessing BP changes (trending ability) rather than estimating absolute BP values. Hence BP changes between all possible pairs of recordings were computed for each patient and no calibration was needed (Fig. 2). A BP change between two recordings *i* and *j* was obtained as: ∆BP(*i*,*j*) = BP(*j*) − BP(*i*) and done both for BP_inv_ and BP_PPG,_ resulting in a list of {∆BP_inv_, ∆BP_PPG_} data pairs for analysis.

To assess the blood pressure trending ability of OptiBP, we used the four-quadrant (4Q) plot method conjointly with polar plots as proposed by Critchley et al. [33, 34]. In the 4Q-method, the upper-right and down-left quadrants contain all {∆BP_inv_, ∆BP_PPG_} pairs showing a concordant direction of change. Hence, the derived concordance rate (CR) represents the percentage of data points in which ∆BP_PPG_ and ∆BP_inv_ change in the same direction. Although the 4Q-method is a good mean to assess trending ability it does not allow to realize the magnitude of changes between {∆BP_inv_, ∆BP_PPG_} pairs. To that end, Critchley suggested to transpose the Cartesian coordinate of the 4Q plots to polar coordinates in so-called polar plots, which enable a quantitative assessments of trending ability. As suggested by the author, we assessed the angular concordance rate at ± 30°, with upper radial limits of ± 5° (mean polar angle) as acceptance limits. To exclude non-significant changes, central exclusion zone of 15% was used for 4Q analysis [33].

The second part of our analysis aimed to pass a clinical judgement on the agreement between BP_inv_ and BP_PPG_. To this end, we used and adapted Saugel et al. [35] BP error-grid analysis which defined five risk zones for a BP measurement method based on twenty-five international specialists in anesthesiology and intensive care medicine. Note that this error-grid was first stratified for critical care and perioperative purpose, hence DBP was deliberately excluded due to its minor role as an isolated value in this setting. Saugel defined these five risk zones (A: no risk to E: dangerous risk) as follow: (A) No risk (i.e., no difference in clinical action between the reference and test method), (B) Low risk (i.e., test method values that deviate from the reference but would probably lead to benign or no treatment), (C) Moderate risk (i.e., test method values that deviate from the reference and would possibly lead to unnecessary or missed treatment with moderate non-life-threatening consequences for the patient), (D) Significant risk (i.e., test method values that deviate from the reference and would lead to unnecessary or missed treatment with severe non-life-threatening consequences for the patient), (E) Dangerous risk (i.e., test method values that deviate from the reference and would lead to unnecessary or missed treatment with life-threatening consequences for the patient). Note that this methodology is based on comparison between absolute BP values and in absence of calibration in our setting, we had to transform them into absolute values by calibrating (i.e., adding an appropriate offset) BP_PPG_ by the average of all BP_inv_ values. By doing so, we artificially find good agreement between BP_PPG_ and BP_inv_ values for patients were there is low BP variability during the measurements. For this reason, all the measurements of patients for whom no significant {∆BP_inv_, ∆ BP_PPG_} pair was found were entirely rejected for this part of the analysis, thus leaving us only with patients with significant BP variability, thereby providing a more realistic evaluation of the performance of our method.

The last part of our analysis aimed at assessing the ability of OptiBP to accurately estimate BP. Each calibrated BP_PPG_ value was compared to its corresponding BP_inv_ value in terms of accuracy (bias) and precision of agreement (SD of the differences) based on the ISO 81060-2:2018 norm [30]. Due to the absence of an applicable norm for continuous BP measurement devices, the latter was used as a point of comparison. When using invasive continuous data as BP reference, our analysis takes into account the variability of said reference when evaluating the agreement with the device under test. More specifically, as illustrated in the right-hand side of Fig. 2, the ISO 81060-2:2018 standard details that if the BP of the device under test falls within the ± 1 SD interval around the average value of BP_inv_, the error is considered to be zero (zero-zone). In addition to providing the accuracy (bias) and precision of agreement (SD) in mmHg, we also provided them as percentage errors, i.e., with normalization of the difference between BP_inv_ and BP_PPG_ by the value of BP_inv_.

### Sample size determination

The minimal sample size to detect a change of 5 mmHg with a worst-case expected standard deviation (SD) of the error of 12 mmHg at a 5% significance level with a power of 90% was determined to be 61 (two-sided, one-sample test) [36]. The value of 5 mmHg was chosen because it is below any physiologically expectable 20% change in MBP, whereas the value of 12 was the upper limit of the 95% confidence interval of the SD obtained in a previous PPG-based study by our group during anesthesia induction [32]. Expecting possible dropouts due to the use of a smartphone (generally lower signal quality than standard pulse oximeters and risk of inadequate finger positioning), a security margin was taken, and 121 patients were enrolled.

## Results

**Fig. 3:**
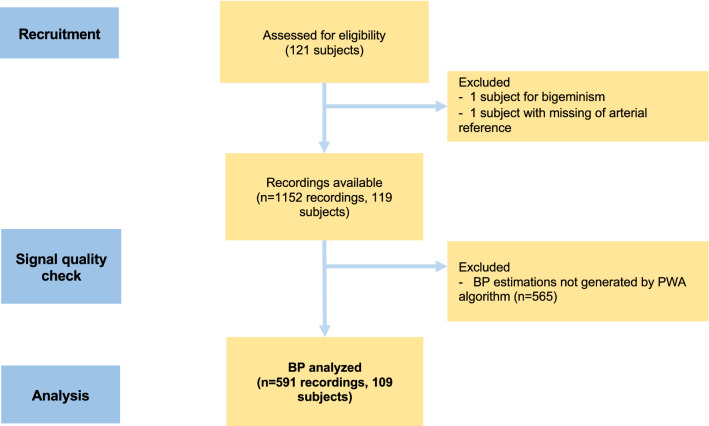
CONSORT Flow Chart of signals used for evaluation. A total of 1152 recordings from 119 patients were analyzed. After exclusion of 2 subjects and rejection of 565 recordings due to BP estimation not generated by the app, we retained 565 recordings from 109 patients (acceptance rate 51.1%)

**Fig. 4:**
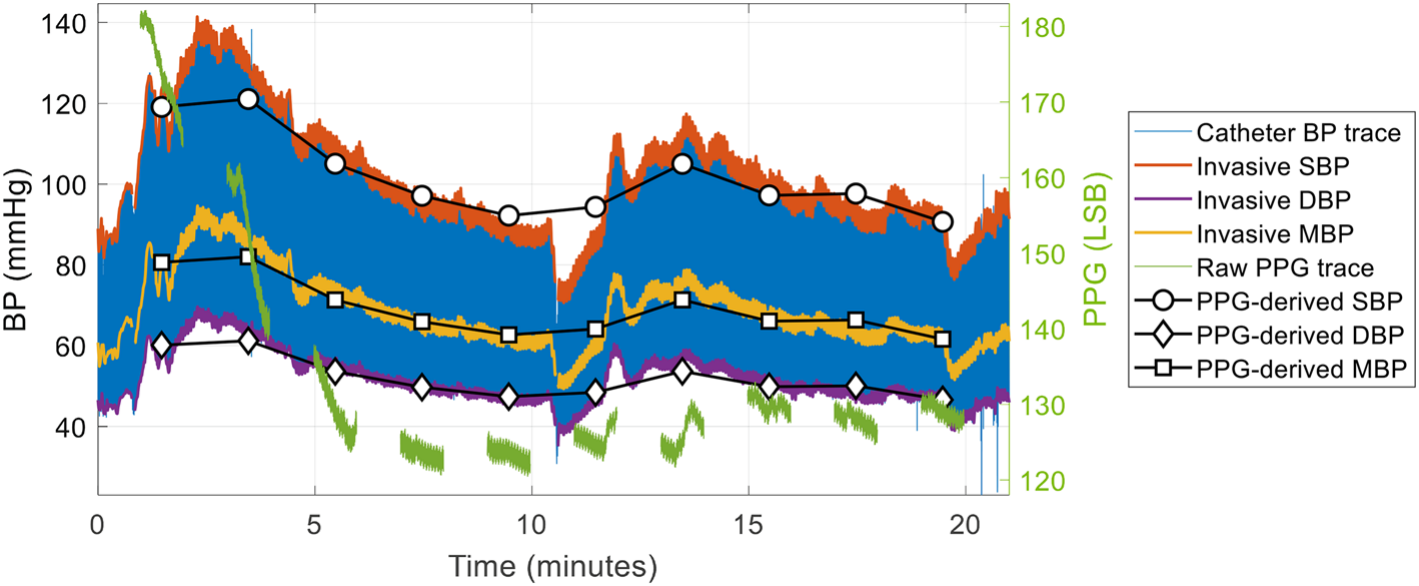
Example of the temporal evolution of invasive blood pressure measurements and blood pressure predictions as generated by the app in a patient. For easy viewing, PPG-derived BP were centered (calibrated) on the average value of the reference in the plot. From the start of the induction (t0), ten continuous invasive BP 1-min segments ( invasive SBP,  invasive DBP,  invasive MBP) aligned in time with ten smartphone recordings ( raw PPG trace) were processed and analyzed by OptiBP algorithm to give PPG-derived SBP, DBP and MBP signals. *BP* blood pressure; *PPG* photoplethysmography; *SBP*, *DBP*, *MBP* systolic, diastolic, mean blood pressure

Recordings from 109 subjects were retained for analysis after exclusion of two subjects and rejection of 565 recordings due to BP estimation not generated by the app (corresponding to a global acceptance rate of 51.1%) (see CONSORT, Fig. 3). Mean age was 58.5 (SD 14.2) with a male/female ratio of 58/51. Vasopressors used for hemodynamic support during the 20 min study periods are summarized in Table 1. The distribution of demographic and biometrics data is summarized in Table 2. Per-patient BP average and mi-max range over the cohort is summarized in Table 3. A typical temporal evolution of BP during anesthesia induction phase in a patient as well as BP estimation by the app is illustrated in Fig. 4.

**Table 1:** Demographic and biometric characteristics of the 109 patients

Age (years)	58.9 ± 14.2 [24–87]
Height (cm)	170.1 ± 8.9 [143–190]
Weight (kg)	76.8 ± 17.1 [45–152]
Body Mass Index (kg/m^2^)	26.5 ± 5.4 [16.5–47.4]
Sex (male/female)	58/51
Active smoking	31 (28.7)
ASA physical status (I/II/III)	1 (0.9)/76 (69.7)/32 (29.4)
*Type of surgery*	
ENT surgery	29 (26.6)
Neurosurgery	66 (60.6)
Spinal surgery	14 (12.8)
*Comorbidities*	
Arterial hypertension	42 (26.6)
Coronary artery disease	14 (12.8)
Arteriopathy	6 (5.5)
Valvular heart disease	10 (9.2)
Renal insufficiency	9 (8.3)
Diabete mellitus	5 (4.6)
Dyslipidemia	21 (19.3)
*Anti-hypertensive medication*	
Beta-blockers	20 (18.3)
ACE inhibitors and ARBs	34 (31.2)
Calcium channel blockers	10 (9.2)
Thiazide diuretics	9 (8.3)
Spirinolactone	6 (5.5)

Data are presented as mean ± SD [range] or count (%)

*ASA* American Society of Anesthesiologists; *ENT* ear, nose, throat; *ACE* angiotensin-converting enzyme; *ARBs* angiotensin II receptor blockers

**Table 2:** Vasopressors used during the 20 min study periods

Type	n	Per patient doses
Ephedrine (mg)	40 (36.7)	15.7 ± 11.8 [5–50]
Phenylephrine (ug)	23 (21.1)	376.1 ± 248.6 [50–900]

Data are presented as mean ± SD [range] or count (%). n represent the number of patients who required vasopressor administration

**Table 3:** Per-patient BP average and min–max range over the cohort

Per-patient SBP average	119 ± 19 [87–173]
Per-patient DBP average	61 ± 9 [37–88]
Per-patient MBP average	81 ± 12 [52–122]
Per-patient SBP min–max range	56 ± 30 [5–167]
Per-patient DBP min–max range	24 ± 13 [2–66]
Per-patient MBP min–max range	36 ± 20 [3–110]

Data are presented as mean ± SD [range]. The average blood pressure (BP) and variability values are the per-patient average value and per-patient minimal–maximal range of invasive BP throughout the entire recording

*SBP* systolic blood pressure; *MBP* mean blood pressure; *DBP* diastolic blood pressure

### Trending ability

**Fig. 5:**
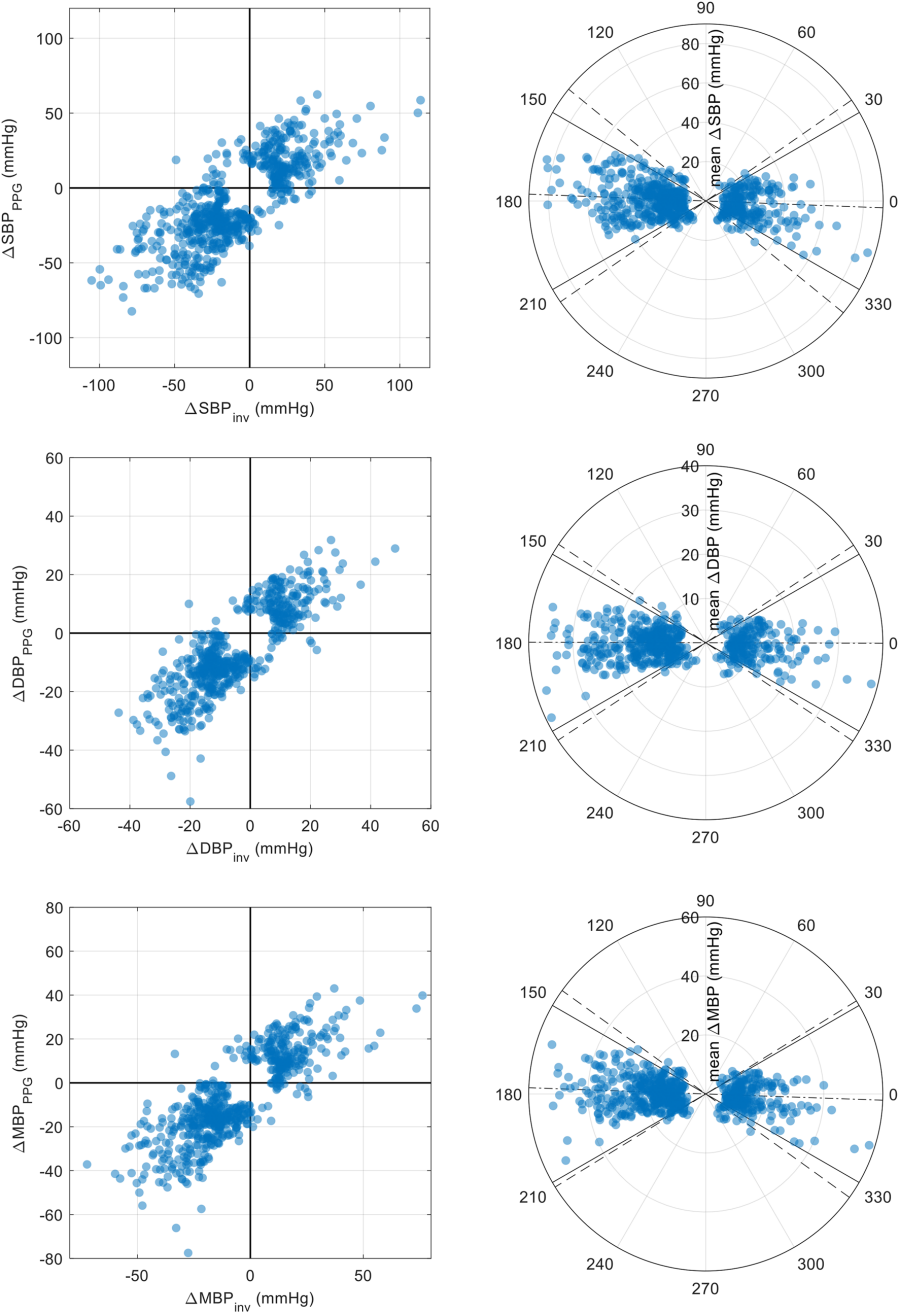
Four-quadrant plots (left) and polar plots (right) for SBP, DBP and MBP depicting the relationship between ∆BP_inv_ and ∆BP_PPG_ at a cohort-wise level. In the polar plots, the distance from center (radial distance) represents the mean of ∆BP_inv_ and ∆BP_PPG_. The solid black lines represent the ± 30° radial limits. The dash-dotted line shows the mean polar angle (angular bias) whereas the two dashed lines represent the 95% confidence interval. Formulae for the polar coordinates, which differ from standard Cartesian-polar conversion, are described in Critchley et al. [33]. *SBP* systolic blood pressure; *MBP* mean blood pressure; *DBP* diastolic blood pressure; *BP*_*inv*_ BP as assessed by the invasive method; *BP*_*PPG*_ BP as assessed by the non-invasive (PPG) method; *∆BP* change in BP of at least ± 15%

Figure 5 shows four-quadrant and polar plots for SBP, DBP and MBP, depicting the relationship between ∆BP_inv_ and ∆BP_PPG_ at a cohort-wise level. When evaluating the trending ability on all changes of more than 15% (corresponding to ~ 34% of all changes), CR values of 91%, 93% and 95% were found for SBP, DBP and MBP, respectively. Polar plots demonstrate an angular CR of 90%, 92% and 92% for SBP, DBP and MBP respectively. Results of polar plots analysis are summarized in Fig. 5 and Table 4.

**Table 4:** Polar plots analyses for SBP, DBP and MBP

	Radial bias (°)	Radial SD (°)	Radial limits of agreement (95%)	Angular CR (± 30°) (%)
SBP	− 2.2	18.8	[− 39.1°, 34.7°]	90
DBP	− 0.2	17.1	[− 33.8°, 33.4°]	92
MBP	− 2.0	17.2	[− 35.8°, 31.7°]	92

*SBP* systolic blood pressure; *MBP* mean blood pressure; *DBP* diastolic blood pressure; *CR* concordance rate

### Error-grid analysis

**Fig. 6:**
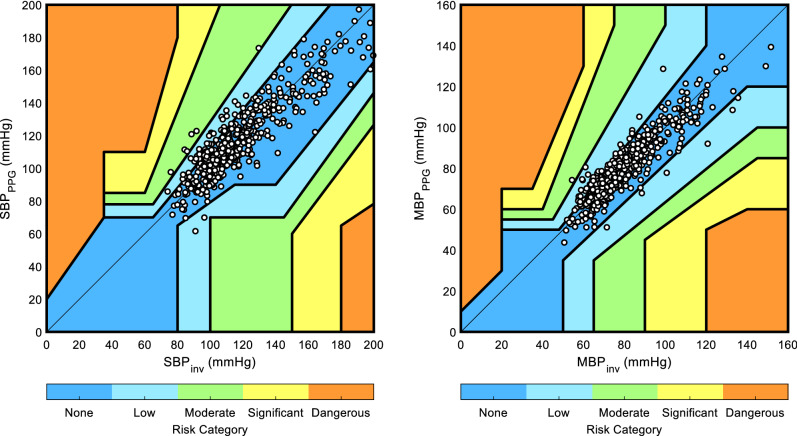
Error grids for systolic and mean blood pressure from 79 (for SBP) and 81 (for MBP) patients with significant intra-subject BP variability. The grid is divided into five zones representing degrees of clinical risks based on the concordance between BP_inv_ and BP_PPG._ Zone A represents no risk for the patient; zone B represents low risk; zone C represents moderate risk; zone D represents significant risk; zone E represents dangerous risk. A–E are 89.8% (n = 449), 9% (n = 45), 1.2% (n = 6), 0%, 0% for SBP and 89.9% (n = 457), 9.8% (n = 50), 0.2% (n = 1), 0%, 0% for MBP. *n* measurement pairs; *SBP* systolic blood pressure; *MBP* mean blood pressure; *BP*_*inv*_ BP as assessed by the invasive method; *BP*_*PPG*_ BP as assessed by the non-invasive (PPG) method

The number of patients with at least one significant {∆BP_inv_, ∆ BP_PPG_} pair—and therefore used for error-grid analysis—was 79 for SBP and 81 for MBP. Error grid analysis showed that the proportions of risk zones A–E were 89.8% (n = 449), 9% (n = 45), 1.2% (n = 6), 0%, 0% for SBP and 89.9% (n = 457), 9.8% (n = 50), 0.2% (n = 1), 0%, 0% for MBP. Continuous error grids are shown in Fig. 6.

### Precision and accuracy

When evaluating the accuracy and precision between BP_inv_ and BP_PPG_ we found a difference [bias ± SD (95% Limits of Agreements)] of 0.0 ± 7.5 mmHg [− 14.9, 14.8], 0.1 ± 2.9 mmHg [− 5.5, 5.7], and 0.1 ± 4.2 mmHg [− 8.3, 8.4] for SBP, DBP and MBP respectively. Expressed as percentage errors, these figures became 0.3 ± 6.6 [− 12.6, 13.3], 0.3 ± 4.9 [− 9.4, 10.0], and 0.3 ± 5.4 [− 10.3, 10.9], respectively.

## Discussion

In this study we were able to evaluate the trending ability of a Pulse Wave Analysis method included in a smartphone application measuring blood pressure compared to an invasively acquired values in highly dynamic conditions. With an angular bias < 5° for SBP, DBP and MBP, our app demonstrates no bias on estimating the amplitude of BP changes and a good trending ability in term of direction of change. Considering magnitude of change, most of the measurements (90–95%) are in the same radial limit of agreements range as those defined by Critchley et al. for the cardiac output (± 30°).

Studies comparing accuracy and precision of continuous non-invasive arterial pressure monitoring systems with invasive arterial pressure measurements in the operating room and critical care settings have led to poor accuracy and precision as reported by Kim et al. [37]. Surprisingly, the use of “zero-zone” is constantly missing in any of the studies reviewed in their meta-analysis which can explain such results. Hence, Kim rightly emphasizes the observed heterogeneity and the lack of consistency in the way acceptability of these devices is defined, throwing a spanner in the works to adopt more specific standards to conduct and report method-comparison studies. Our study was designed to consider such “zero-zone”. Multiple blood pressure measurement per individual time point being technically not feasible we followed Hapfelmeier et al. and comply with their recommendations [38]. To that end, the amplitude of the ∆BP_PPG_ changes was compared to that of the ∆BP_inv_ changes in terms of accuracy (bias) and precision of agreements (SD of the differences) based on the ISO 81060-2:18 norm [30]. Based on the “zero zone” methodology, our approach showed promising accuracy and precision. Although we are aware that it is very difficult to draw any conclusions in the light of our results, we are sure it will bring a good point of comparison with other studies. Hence, to ensure reproducibility, we also reported values in percentage error as proposed in [38].

We choose to transpose Saugel et al. [35] error grid to our specific use case to assess the clinical relevance of the BP measurement differences between our app and the intra-arterial reference. In contrast with Bland–Altman analysis which only assesses statistical agreement between the test and reference method for BP monitoring, Saugel make a step over deriving the error grid analysis—first use for glucose measurement analysis—to BP measurements to combine clinical concordance to therapeutic consequences. After exclusion of patients with low BP variability, our app demonstrates a good clinical concordance with more than 98% of measurement pairs in no- or low-risk zones for SBP and MBP, of which more than 89% in no-risk zone. These results are better than those presented by Takashi et al. [39] which investigated confounding factors affecting discrepancies between invasive arterial pressure (IAP) measurement and non-invasive blood pressure (NIBP) oscillometric technique using error grid analysis in 100 patients undergoing general anesthesia. In their study, difference between IAP and NIBP for MBP (not SBP) were not clinically acceptable. After multiple regression analysis, continuous phenylephrine administration (more than half of the operation time) and age > 60 years old were the major factors for an increased clinical risk of IAP and NIBP discrepancies which—as mentioned by the author—is consistent with other studies in the field. We did not specifically design our study to investigate potential cofounders but even after analyzing MBP sub-group in the study by Takashi et al. and rejection of patients with phenylephrine administration, our obtain a higher clinical concordance than standard NIBP oscillometric measurement. This difference can be explained in part by a different median age between our study and that of Takashi of 58.9 vs 68 respectively. Although this last result must be taken with caution due to major difference in the two studies stratification it raises an important question. Association for the Advancement of Medical Instrumentation (AAMI) approach requires hemodynamic stability for NIBP to deliver reliable values. This questions the methodology or statistical analysis to use NIBP in highly dynamic blood pressure conditions which can be easily found when using a gold standard arterial catheter as proposed by the AAMI. Moreover, this open up a wider question, already raised by Fortin et al., about the use of such devices in real life as well as the need for new evaluation standard taking into consideration trending ability of devices under real-life clinical conditions [40].

Our study has several limitations. First, we used polar plots as defined by Critchley et al. which was validated against concordance and the opinion by authors of limited comparatives studies. As suggested by Peyton and Chong [41] guidelines has to be adjusted in the light of the effective reference and/or technique applied to the measurement, even suggesting an increase of radial limits of agreement (RLOA) from 30 to 45%. Although numerous studies [32, 42–45] have used such statistical methodology to validate continuous blood pressure devices, to our knowledge, there is currently no consensus on the limits to be applied [46]. Similar limitations can be reported using error-grid which is in our sense an innovative and promising tool to validate blood pressure devices but currently lacks the hindsight to issue real guidelines adapted to specific populations and reference methods. Secondly, repeated measurements with a stable BP were not possible to determine in such study due to high blood pressure variations. Thus, determining the precision of measurement of our app was not possible. Third, to evaluate clinical risk as defined by error-grid, given the highly dynamic environment, we had to calibrate BP_PPG_ by the average of all BP_inv_ values. This implies a perfect calibration which is not feasible in practice. Finally, we observed a poor—but expected—acceptance rate due to induction context that was not found in the final use-case of the investigated smartphone app [21, 22]. Additionally, no population stratification has been done and so more investigation should be conducted to validate such technology in relevant population.

## Conclusions

The most frequently used home blood patient monitoring system is the oscillometric cuff. Although it brings many advantages, its usage is limited in restrained human resources and infrastructure conditions, leading to less controlled BP values. With six billion mobiles phones users worldwide, our study could lead the way for mobile devices to leverage the monitoring of BP in the near future and impact health assessment capabilities.

To the best of our knowledge, this is the first time a smartphone app was compared to an invasive BP reference. Its trending ability was investigated in highly dynamic conditions, demonstrating high concordance and accuracy. Further studies are needed to assess implementation in clinical practice.
